# Supplementary material for: Parkin is a disease modifier in the mutant SOD1 mouse model of ALS
Source: EMBO Mol Med. 2018 Aug 20;10(10):e8888. doi: 10.15252/emmm.201808888 (PMC6180298; doi:10.15252/emmm.201808888)
Supplement: Supplementary file 1 — Expanded View Figures PDF [file EMMM-10-e8888-s001.pdf]

Expanded View Figures

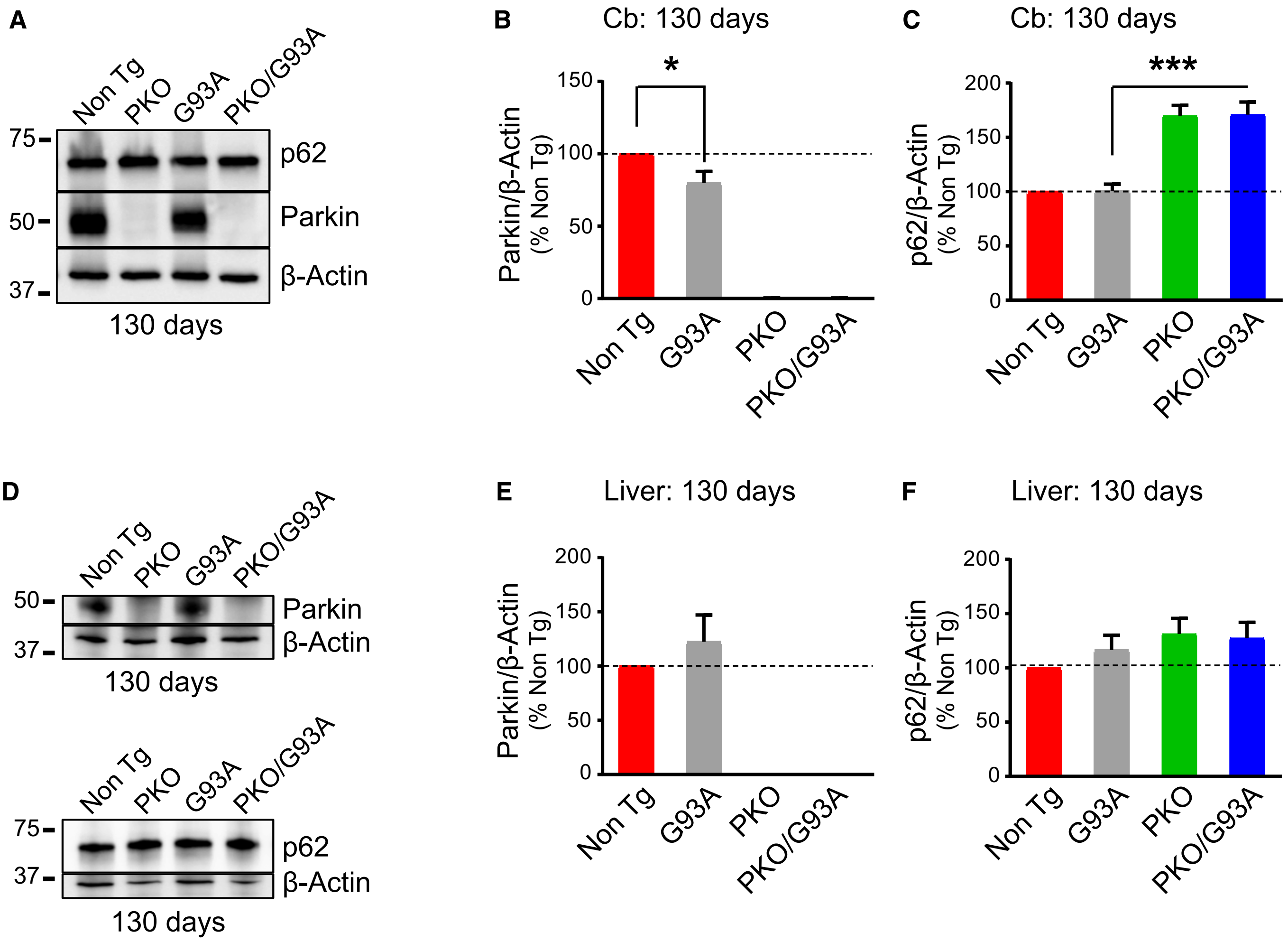

Figure EV1. Parkin protein levels are unaffected in liver while a small decrease is measured in G93A cerebellum.

A Representative Western blot for Parkin and p62 in cerebellum (Cb) homogenates at 130 days.

B Quantification indicates that Parkin is decreased in G93A mice relative to Non Tg at 130 days. Protein levels were normalized by β-actin. Results are expressed as mean ± SEM and percent of Non Tg; n = 8 (four males and four females) mice per group. *P = 0.039 by paired Wilcoxon's test.

C Quantification of p62 in the homogenates indicates that p62 is increased in PKO and PKO/G93A mice, independent of SOD1-G93A expression, at 130 days of age. β-actin was used for normalization. Results are expressed as mean ± SEM and percent of Non Tg; n = 8 (four males and four females) mice per group. No statistically significant differences were found between Non Tg and G93A by paired Friedman's test with Dunn's correction (P = 0.99). ***P = 0.0001 by paired Student's t-test (G93A vs. PKO/G93A).

D Representative Western blot of Parkin and p62 in liver homogenates at 130 days.

E Parkin protein levels were quantified at 130 days. β-actin was used for normalization. Results are expressed as mean ± SEM and percent of Non Tg; n = 8 (four males and four females) mice per group. No statistically significant differences were found between Non Tg and G93A (P = 0.546 by paired Wilcoxon's test).

F Quantification of p62 protein levels in liver at 130 days. Results are expressed as mean ± SEM and percent of Non Tg; n = 8 (four males and four females) mice per group. No statistically significant differences were found between Non Tg and G93A (P = 0.546 by paired Wilcoxon's test). No statistically significant differences were found among the other groups.

Source data are available online for this figure.

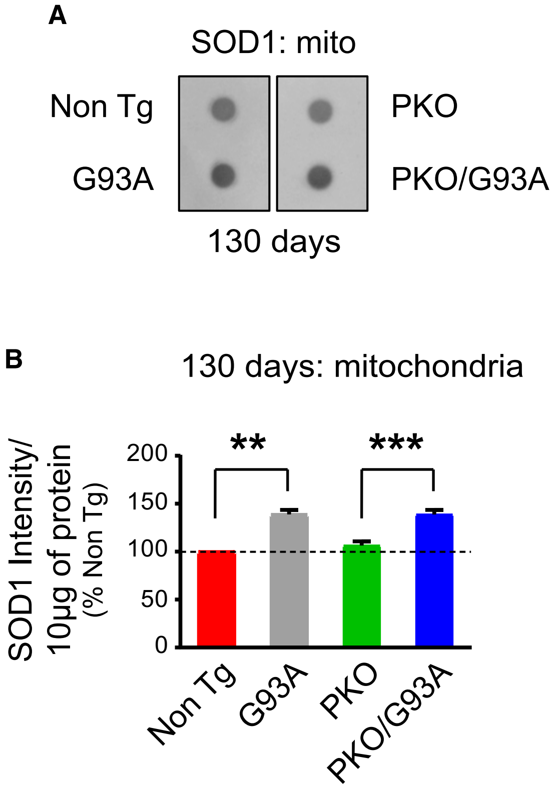

Figure EV2. SOD1 aggregates similarly in spinal cord mitochondria of G93A and PKO/G93A mice.

A Representative filter-trap blot for detection of SOD1 aggregates in spinal cord mitochondria at 130 days.

B Quantification of the SOD1 signal intensity at 130 days of age revealed no statistically significant differences between G93A and PKO/G93A, indicative of similar amount of aggregates in mitochondria. Results are expressed as mean ± SEM and as percent of Non Tg; n = 8 (four males and four females) mice per group. No statistically significant differences were found between G93A and PKO/G93A (P = 0.987 by paired Student's t-test). **P = 0.0058 by paired Friedman's test with Dunn's correction (for Non Tg vs. G93A), ***P = 0.0002, by paired one-way ANOVA with Tukey's correction (for PKO vs. PKO/G93A).

Source data are available online for this figure.

© 2018 The Authors

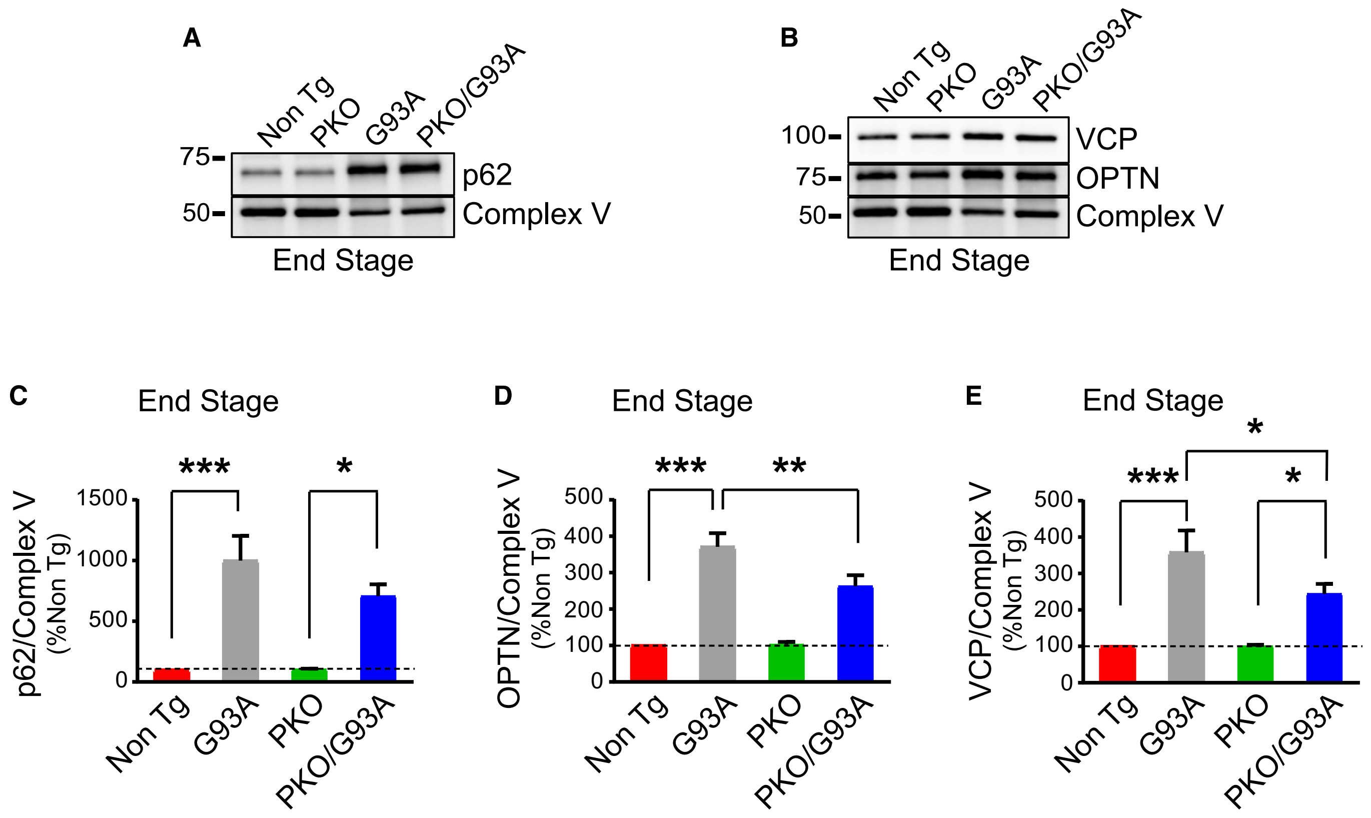

Figure EV3. Parkin knockout mitigates the accumulation of mitophagy adaptors in G93A mitochondria at disease end stage.

A, B Representative Western blots of p62 (A), OPTN, and VCP (B) in spinal cord mitochondria from end-stage mice.

C p62 quantification, using Complex V as normalizer, shows a strong accumulation of p62 in SOD1-G93A mitochondria at end stage. Results are expressed as mean ± SEM and as percent of Non Tg; $n = 8$ (four males and four females) mice per group. No statistically significant differences were found between G93A and PKO/G93A ($P = 0.078$ by paired Wilcoxon's test); ***$P = 0.0007$ (Non Tg vs. G93A) and *$P = 0.037$ (PKO and PKO/G93A) both by paired Friedman's test with Dunn's correction.

D Quantification of OPTN accumulation in end-stage mitochondria, with Complex V as protein loading control. Results are expressed as mean ± SEM and as percent of Non Tg; $n = 8$ (four males and four females) mice per group. **$P = 0.0078$ (for G93A and PKO/G93A) by paired Wilcoxon's test; ***$P = 0.0003$ (Non Tg vs. G93A) by paired Friedman's test with Dunn's correction. No other statistically significant differences were found.

E Quantification of VCP in mitochondria at disease end stage. Complex V was used as normalizer. Results are expressed as mean ± SEM and as percent of Non Tg; $n = 8$ (four males and four females) mice per group. *$P = 0.039$ (for G93A and PKO/G93A) by paired Wilcoxon's test; ***$P = 0.0007$ (Non Tg vs. G93A) and *$P = 0.037$ (PKO and PKO/G93A) both by paired Friedman's test with Dunn's correction.

Source data are available online for this figure.

Figure EV4. Increased mitochondrial protein turnover in G93A mice is attenuated by Parkin knockout.

A, B Western blots of COXI (A) and Tim23 (B) in spinal cord homogenates at 130 days.

C Quantification of COXI at 130 days with β-actin as a normalizer. Results are expressed as mean ± SEM and as percent of Non Tg; $n = 8$ (four males and four females) mice per group. No statistically significant differences were found between G93A and PKO/G93A ($P = 0.493$ by paired Student's t-test).

D Quantification of Tim23 at 130 days, using β-actin as loading control, showed decreased levels of Tim23 in G93A mice. Results are expressed as mean ± SEM and as a percent of Non Tg; $n = 8$ (four males and four females) mice per group. No statistically significant differences were found between G93A and PKO/G93A ($P = 0.921$ by paired Student's t-test). *$P = 0.035$ (Non Tg Vs. G93A) by paired Friedman's test with Dunn's correction. No other statistically significant differences were found.

E, F Representative Western blots of COXI (E) and Tim23 (F) in disease end-stage homogenates.

G Quantification of COXI at disease end stage using β-actin as loading control. Results are expressed as mean ± SEM and as percent of Non Tg; $n = 5$ (three males and two females) mice per group. *$P = 0.047$ for (G93A and PKO/G93A) by paired Student's t-test; **$P = 0.0018$ by paired Friedman's test with Dunn's correction (Non Tg vs. G93A).

H Quantification of Tim23 with β-actin as normalizer at end stage showed that Parkin knockout can alleviate the turnover of mitochondrial proteins in G93A spinal cords. Results are expressed as mean ± SEM and as percent of Non Tg; $n = 8$ (four males and four females) mice per group. No statistically significant differences were found between G93A and PKO/G93A ($P = 0.190$ by paired Student's t-test). *$P = 0.037$ (for PKO vs. PKO/G93A) by paired Friedman's test with Dunn's correction. No other statistically significant differences were found.

Source data are available online for this figure.

▶

© 2018 The Authors

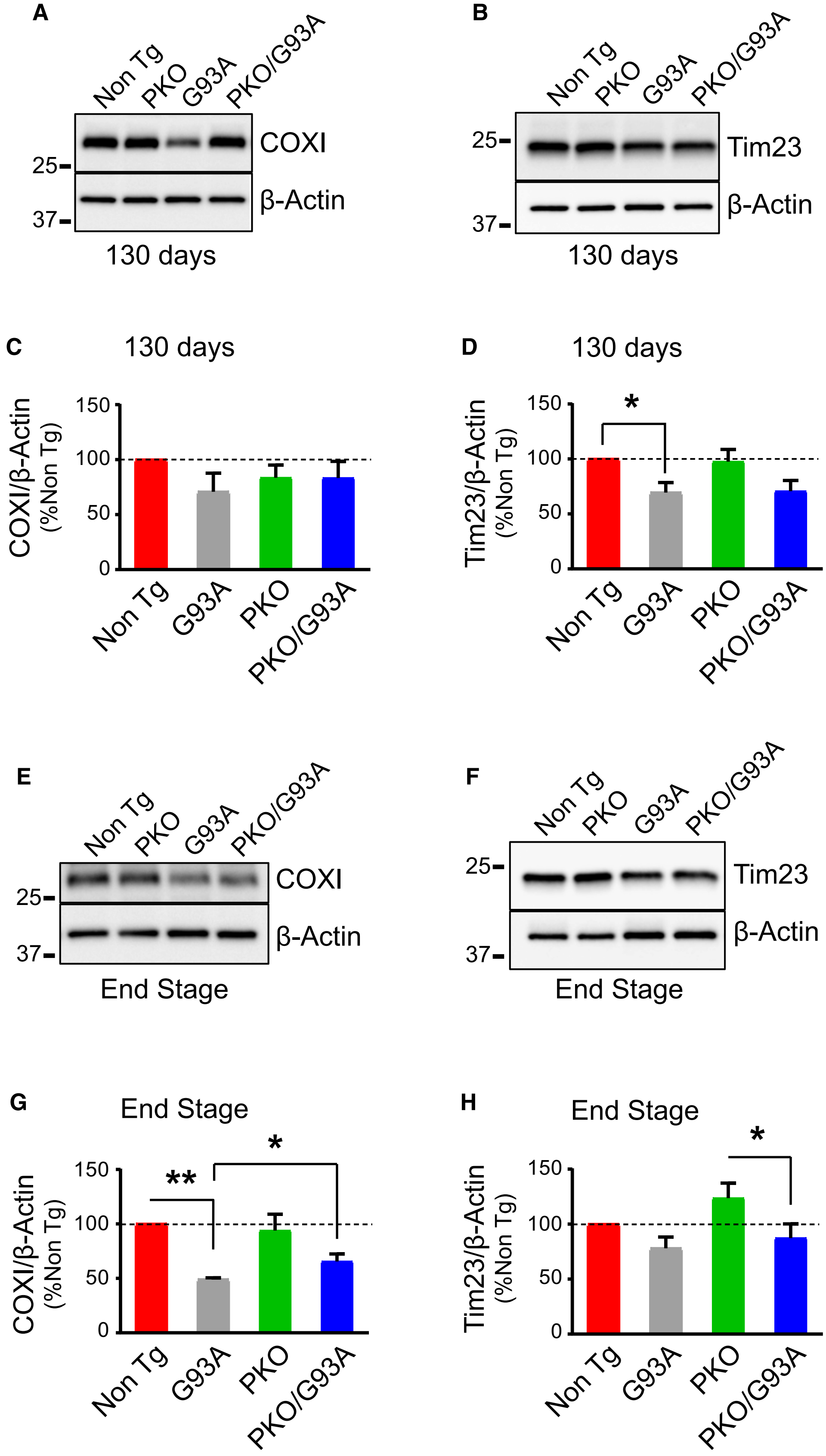

Figure EV4.

© 2018 The Authors

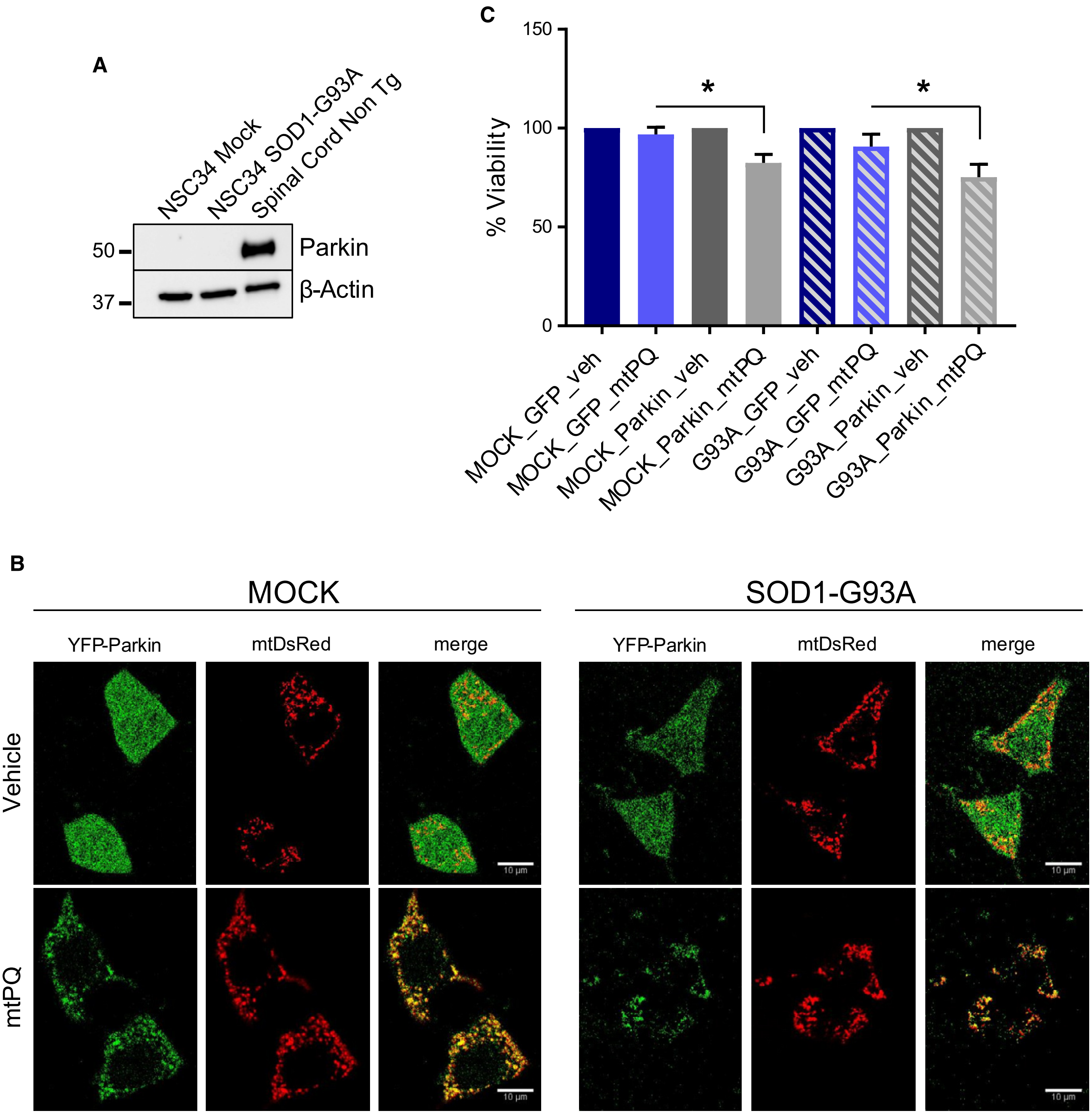

Figure EV5. Parkin overexpression increases vulnerability to mitochondrial oxidative stress in mutant SOD1-expressing NSC34 motor neuron-like cells.

A Western blot of Parkin in lysates from NSC34 motor neuron-like cells, stably transfected with either human SOD1-G93A or empty vector (Mock). Mouse spinal cord homogenate was used as a positive control for Parkin. β-actin was the loading control.

B Representative images of NSC34 cells transfected with mtDsRed and YFP-Parkin or GFP and treated with 10 μM mtPQ or vehicle (DMSO) for 24 h. Scale bar, 10 μm. YFP-Parkin clusters with mitochondria upon oxidative stress.

C Viability of NSC34 cells after transfection with YFP-Parkin or pEGFP-N1 and treatment with DMSO (vehicle) or 10 μM mtPQ for 24 h. Results are expressed as mean ± SEM and as percent of vehicle-treated cells (for each plasmid transfection). n = 40 fields per condition (from two independent experiments); for Mock NSC34 cells, GFP vs. Parkin transfected, treated with mtPQ, *P = 0.013, by unpaired Student's t-test; for G93A NSC34 cells, GFP vs. Parkin transfected, treated with mtPQ, *P = 0.029, by unpaired Mann–Whitney t-test.

Source data are available online for this figure.

© 2018 The Authors
