# Supplementary material for: Parkin is a disease modifier in the mutant SOD1 mouse model of ALS
Source: EMBO Mol Med. 2018 Aug 20;10(10):e8888. doi: 10.15252/emmm.201808888 (PMC6180298; doi:10.15252/emmm.201808888)

Palomo GM et al. Expanded Viev 1

Cerebellum Western blots

Developed with anti-Parkin PRK8

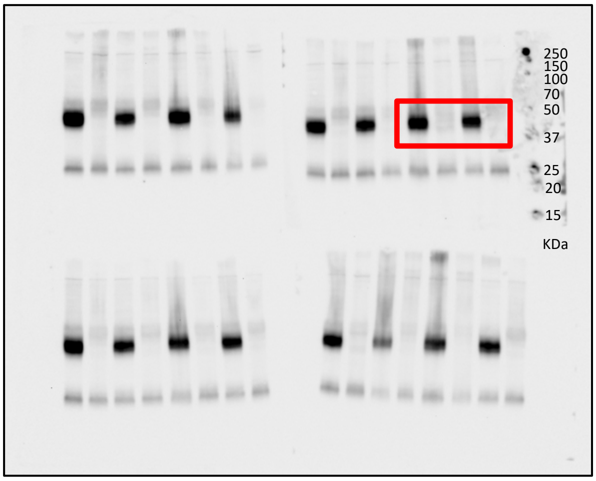

Developed with anti-p62

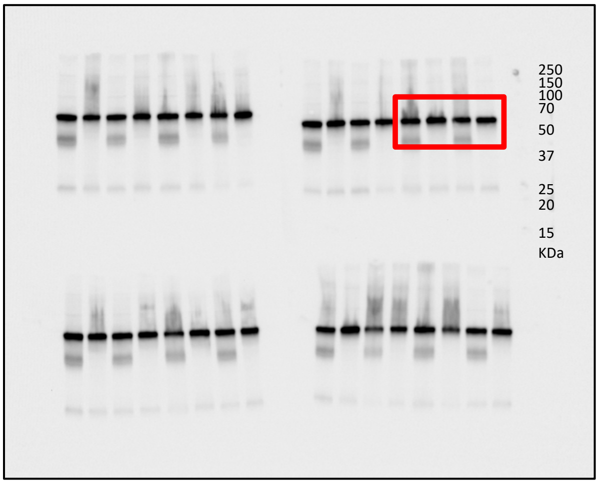

Developed with anti-β-actin

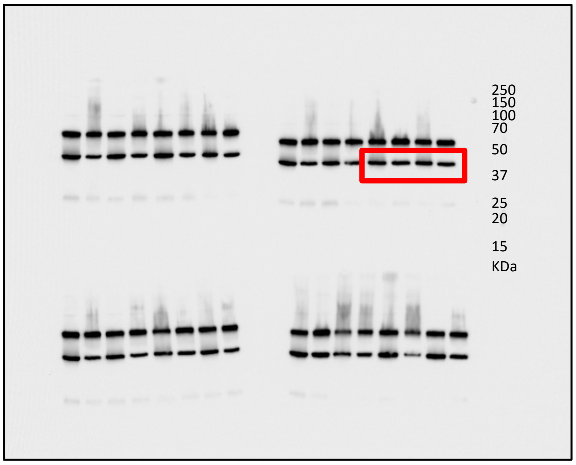

Molecular weight markers

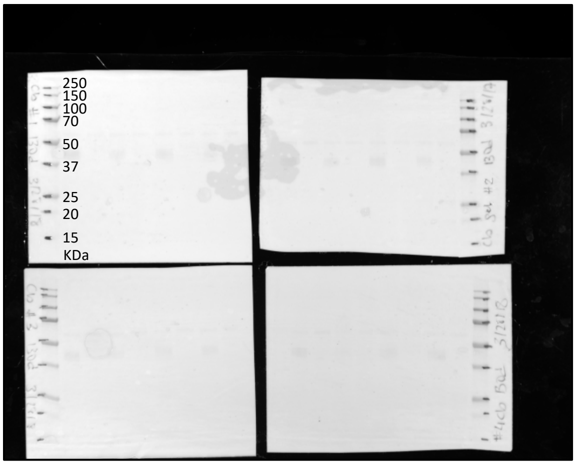

Palomo GM et al. Expanded Viev 1

Liver Western blots

Developed with anti-Parkin PRK8

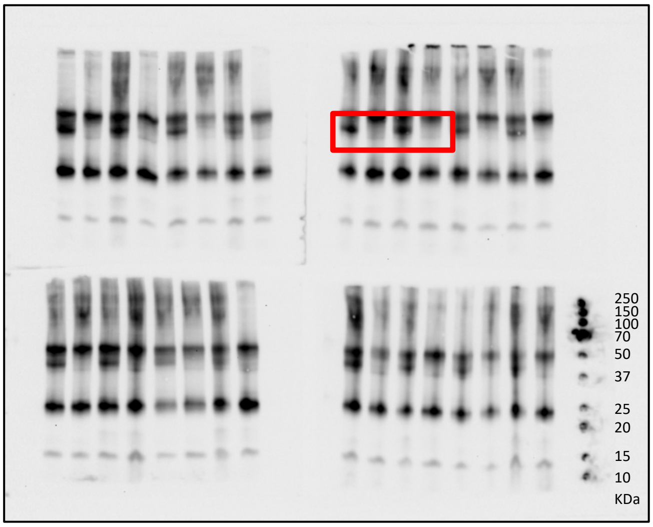

Developed with anti-β-actin

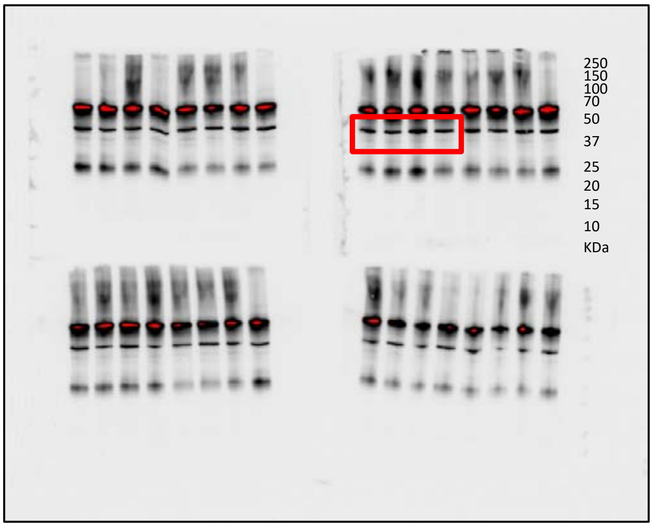

Developed with anti-p62

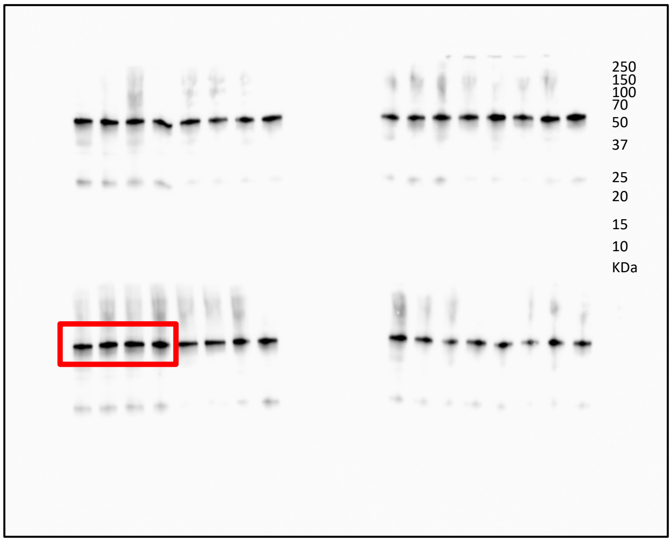

Developed with anti-β-actin

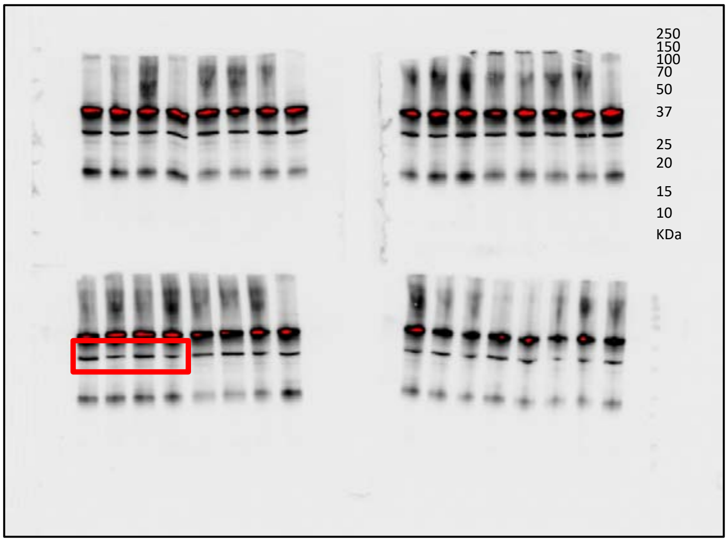

Palomo GM et al. Expanded Viev 1

Liver Western blots

Molecular weight markers

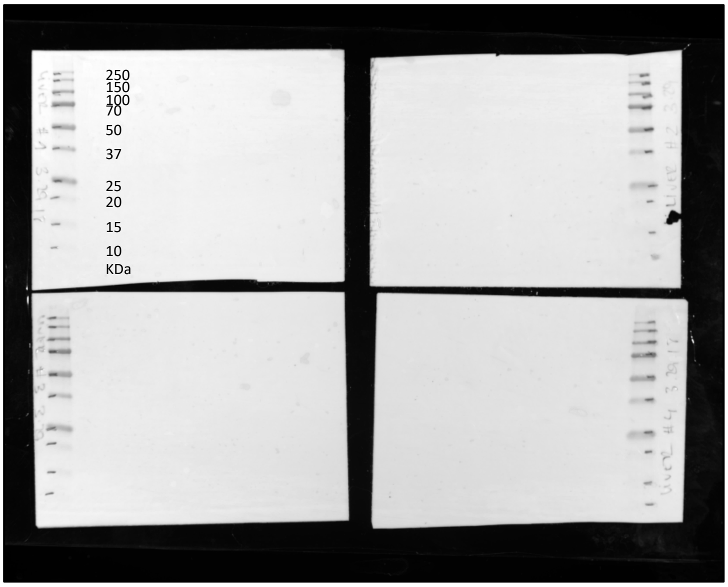

Supplement: Supplementary file 4 — Source Data for Expanded View [file EMMM-10-e8888-s012.zip › EMM_8888_EV_SD/Figure_EV1_SD.pdf]
