# Supplementary figures and images for: Parkin is a disease modifier in the mutant SOD1 mouse model of ALS
Source: EMBO Mol Med. 2018 Aug 20;10(10):e8888. doi: 10.15252/emmm.201808888 (PMC6180298; doi:10.15252/emmm.201808888)

Palomo GM et al. Expanded View 2

Developed with anti-SOD1

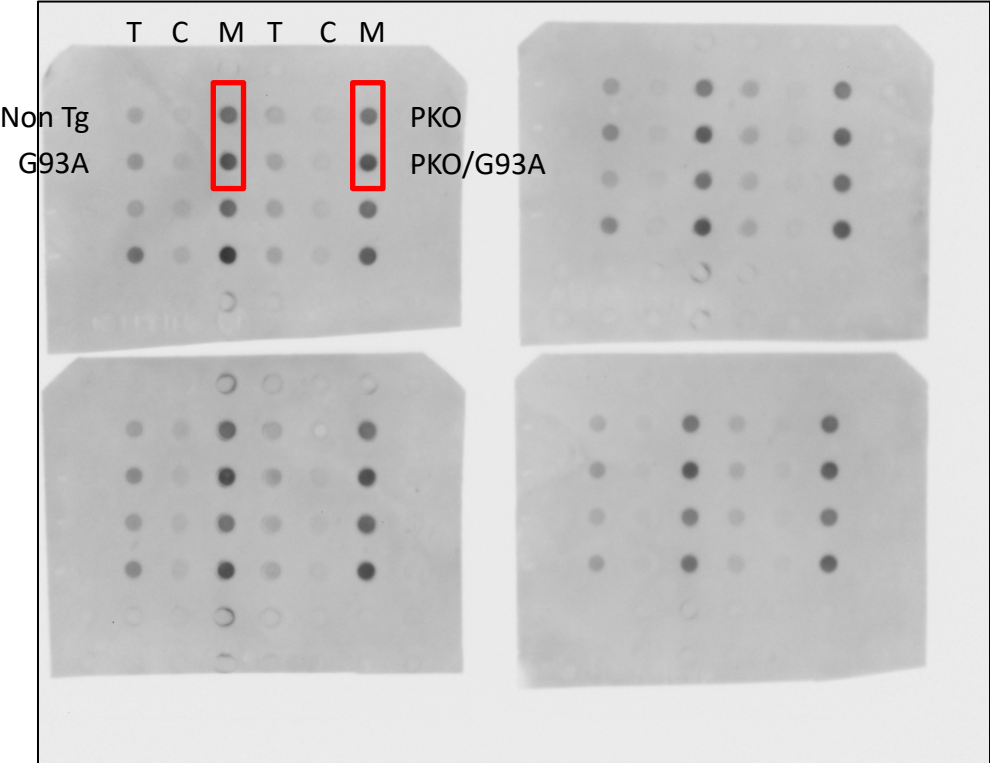

Supplement: Supplementary file 4 — Source Data for Expanded View [file EMMM-10-e8888-s012.zip › EMM_8888_EV_SD/Figure_EV2_SD.pdf]

End Stage

Molecular weight markers for COXI  
membrane

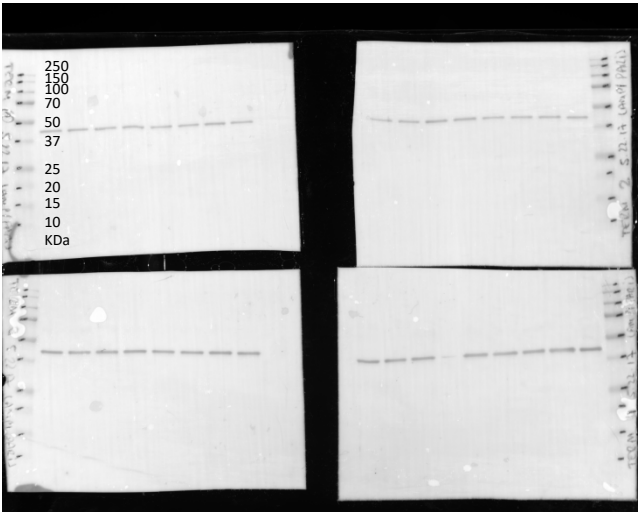

Supplement: Supplementary file 4 — Source Data for Expanded View [file EMMM-10-e8888-s012.zip › EMM_8888_EV_SD/Figure_EV4_SD.pdf]

Developed with anti-Parkin PRK8

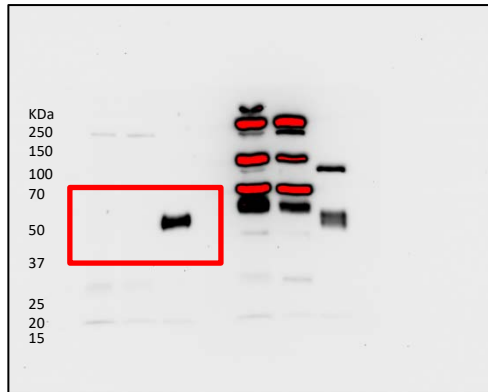

Developed with anti- $\beta$ -actin

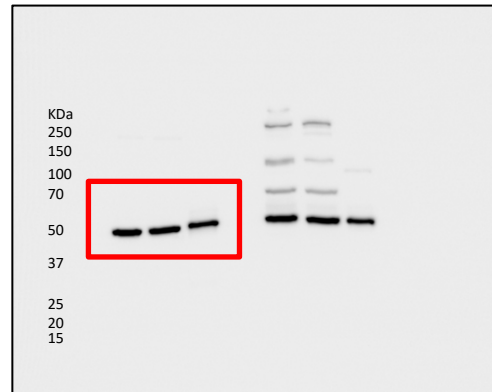

Molecular weight markers

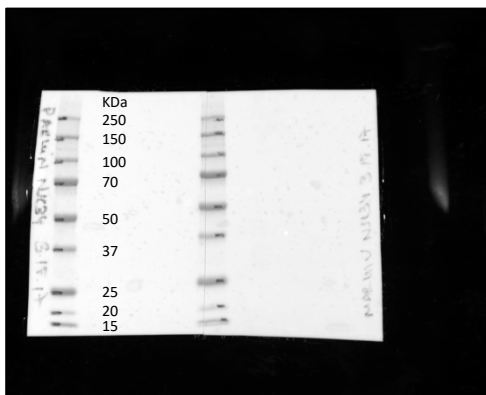

Supplement: Supplementary file 4 — Source Data for Expanded View [file EMMM-10-e8888-s012.zip › EMM_8888_EV_SD/Figure_EV5_SD.pdf]

Palomo GM et al. Figure 6

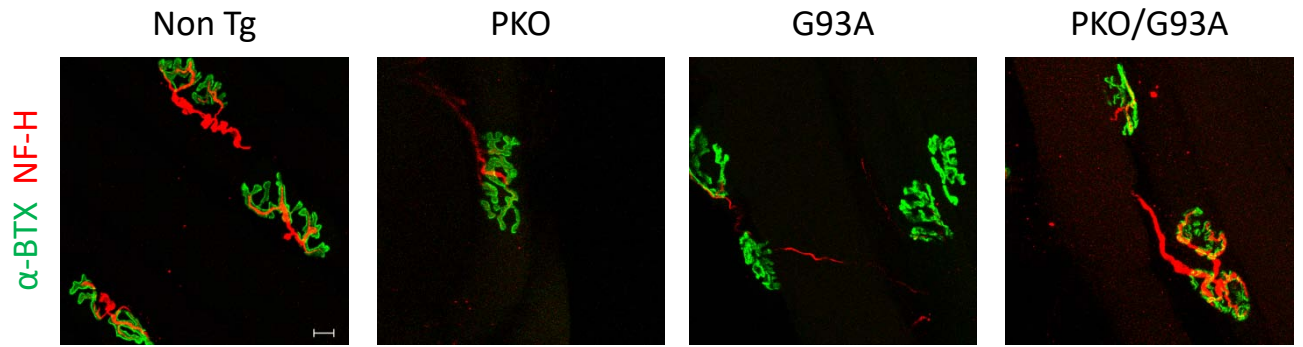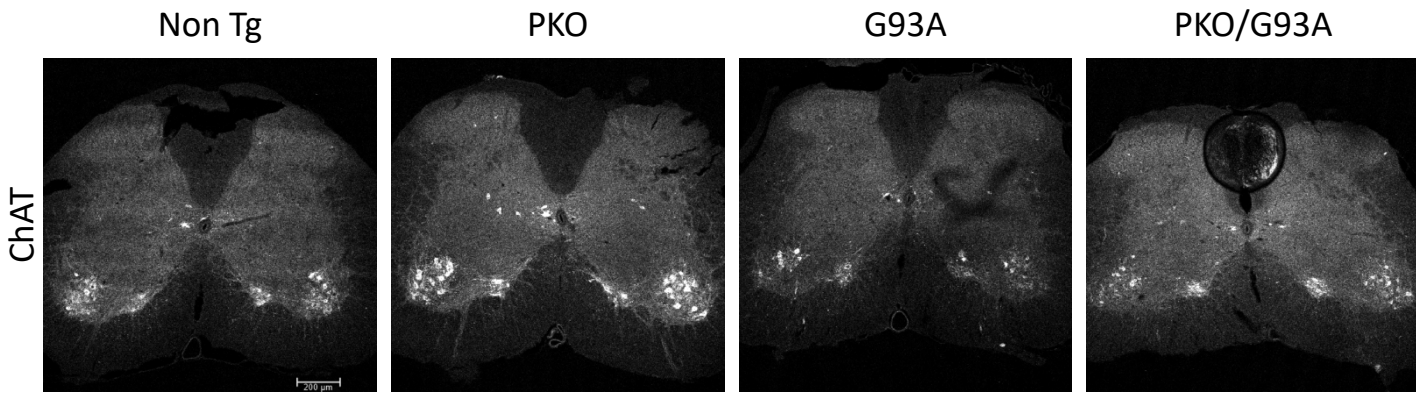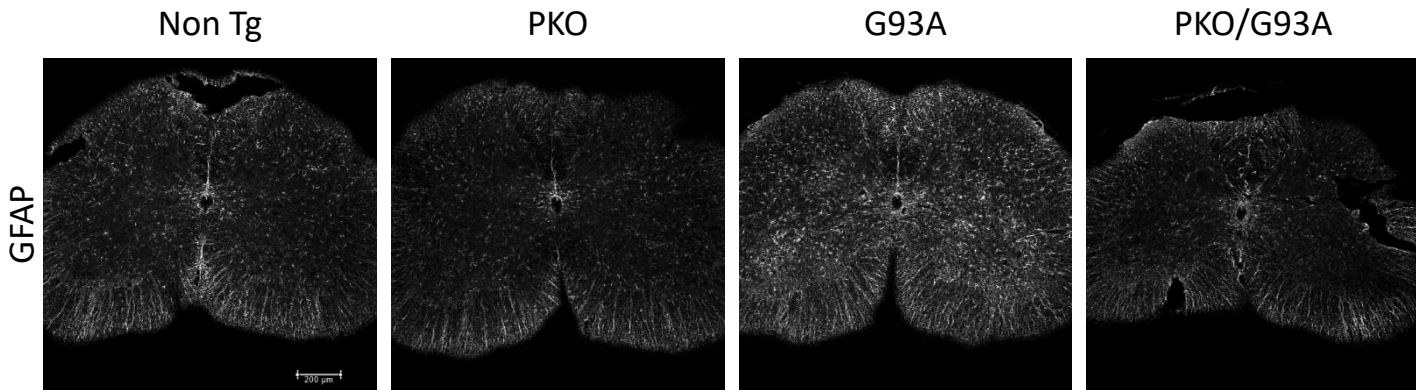

Supplement: Supplementary file 11 — Source Data for Figure 6 [file EMMM-10-e8888-s009.pdf]

Developed with anti-PARIS

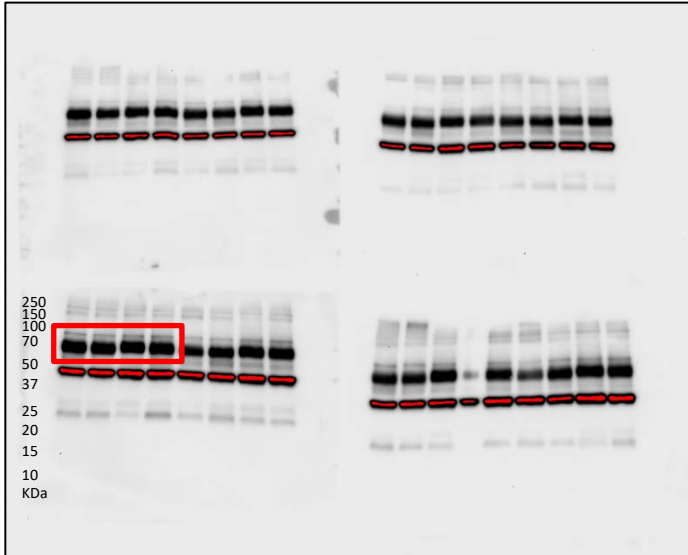

Developed with anti- $\beta$ -actin

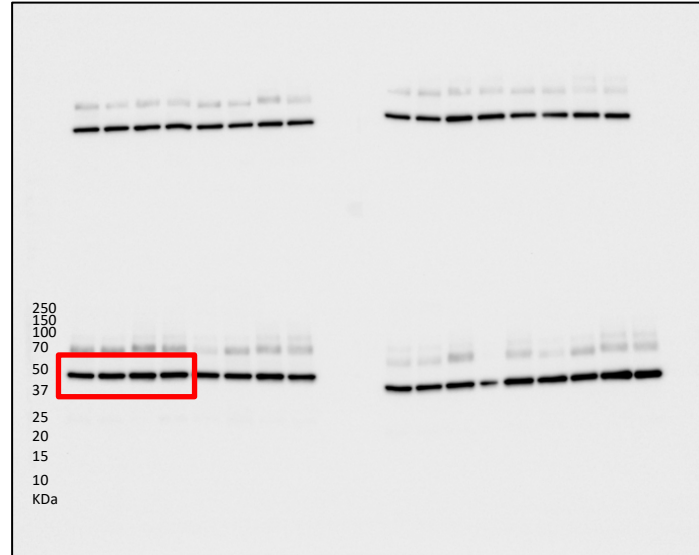

Molecular weight markers

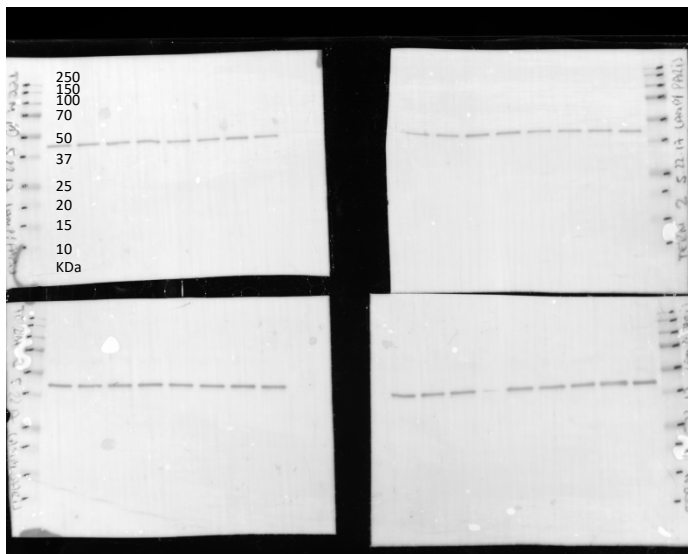

Supplement: Supplementary file 12 — Source Data for Figure 7 [file EMMM-10-e8888-s010.pdf]
