# Supplementary material for: Parkin is a disease modifier in the mutant SOD1 mouse model of ALS
Source: EMBO Mol Med. 2018 Aug 20;10(10):e8888. doi: 10.15252/emmm.201808888 (PMC6180298; doi:10.15252/emmm.201808888)

Palomo GM et al. Figure 3

Developed with anti-Parkin PRK8

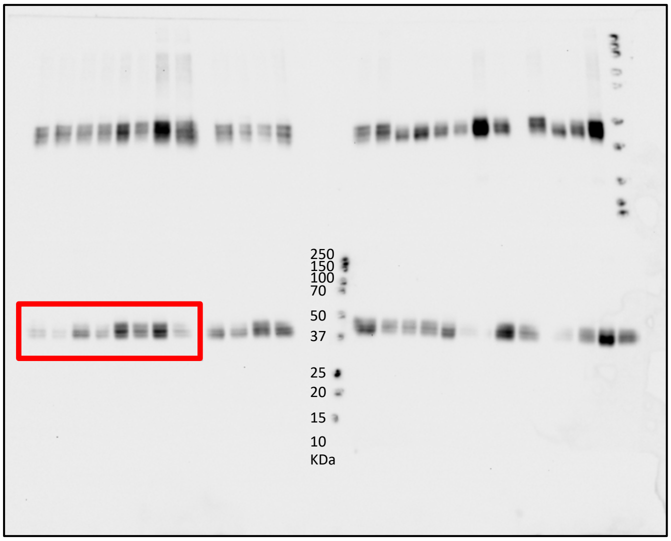

Developed with anti-β-actin

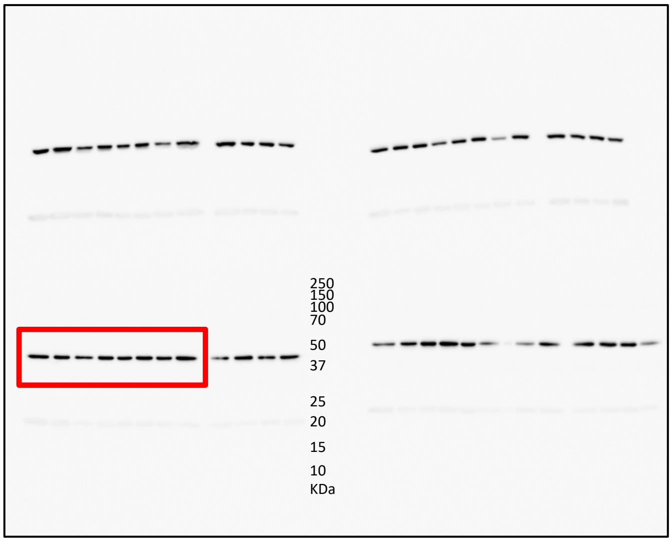

Developed with anti-Parkin PRK8

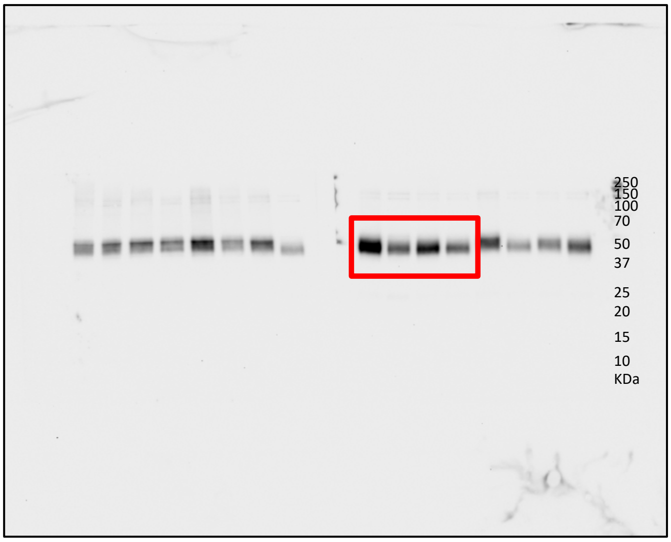

Developed with anti-Complex V

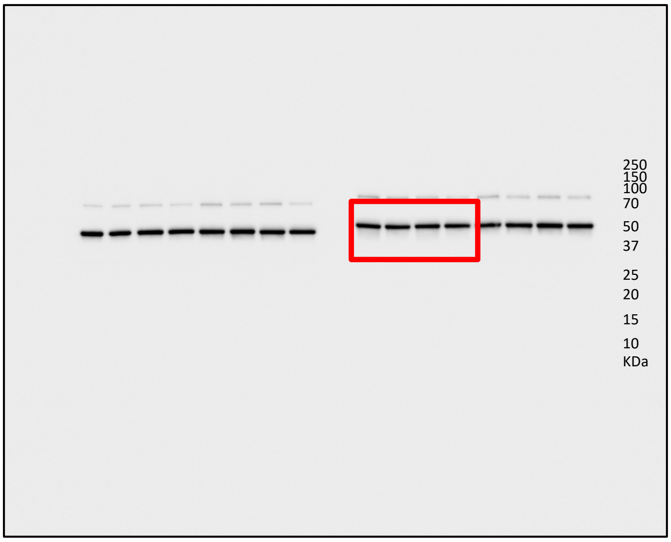

Palomo GM et al. Figure 3

Molecular weight markers

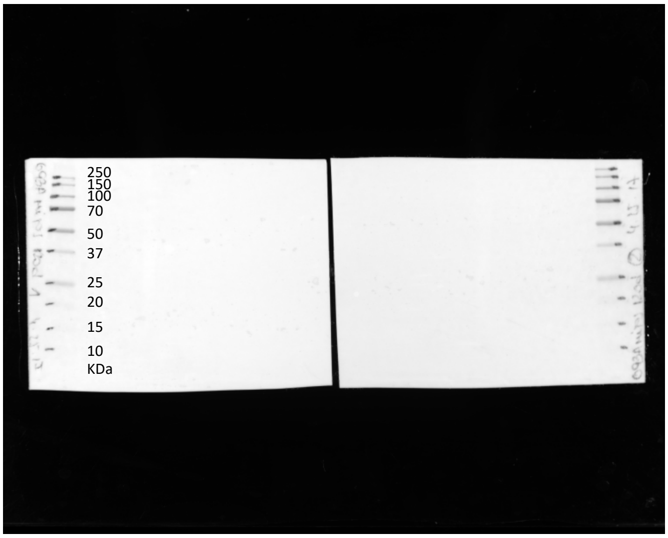

Supplement: Supplementary file 8 — Source Data for Figure 3 [file EMMM-10-e8888-s006.pdf]
