# Supplementary material for: Parkin is a disease modifier in the mutant SOD1 mouse model of ALS
Source: EMBO Mol Med. 2018 Aug 20;10(10):e8888. doi: 10.15252/emmm.201808888 (PMC6180298; doi:10.15252/emmm.201808888)

Palomo GM et al. Figure 4

Developed with anti-Parkin PRK8

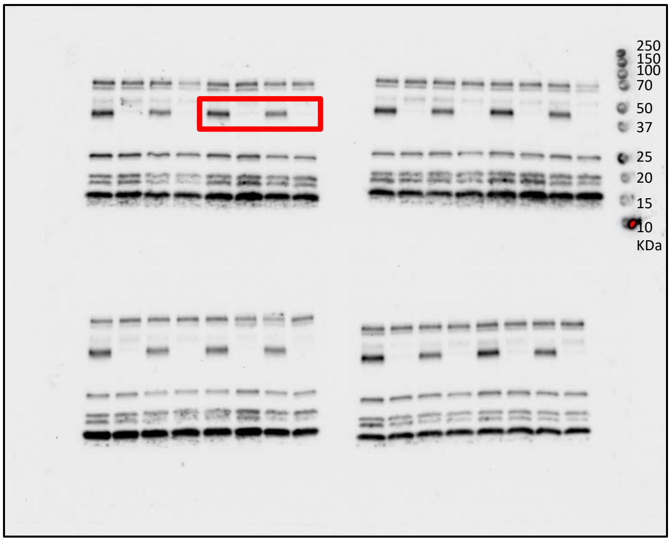

Developed with anti-SOD1

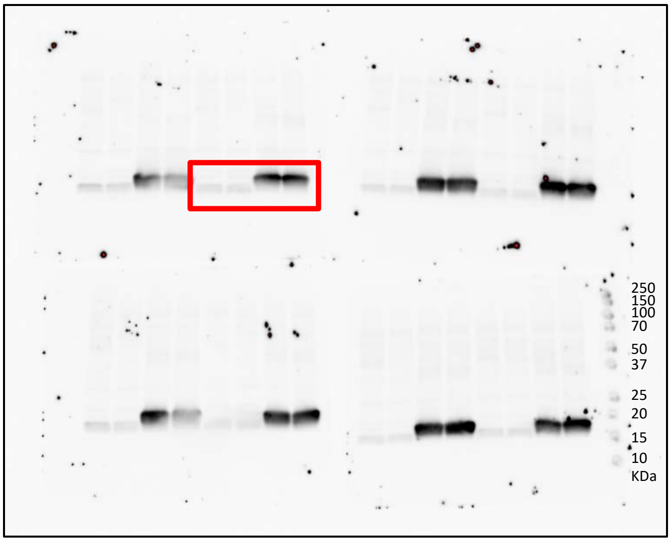

Developed with anti-β-actin

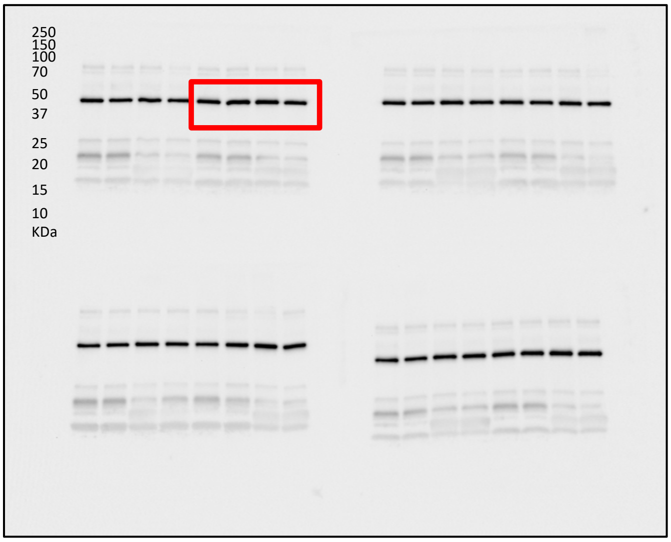

Molecular weight markers

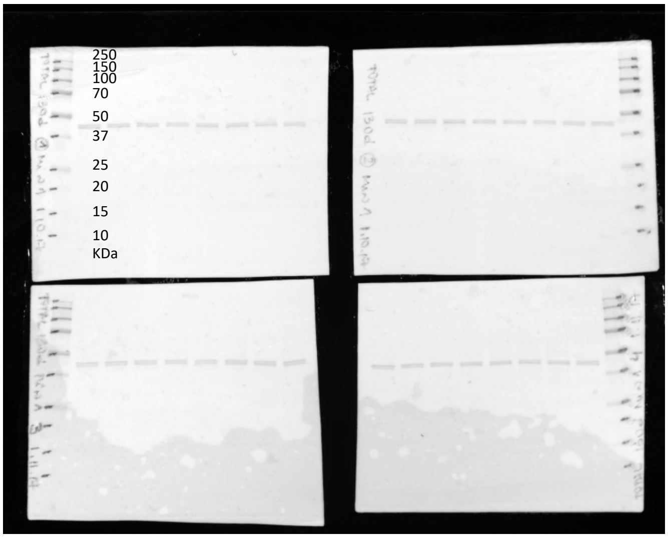

Palomo GM et al. Figure 4

Developed with anti-SOD1

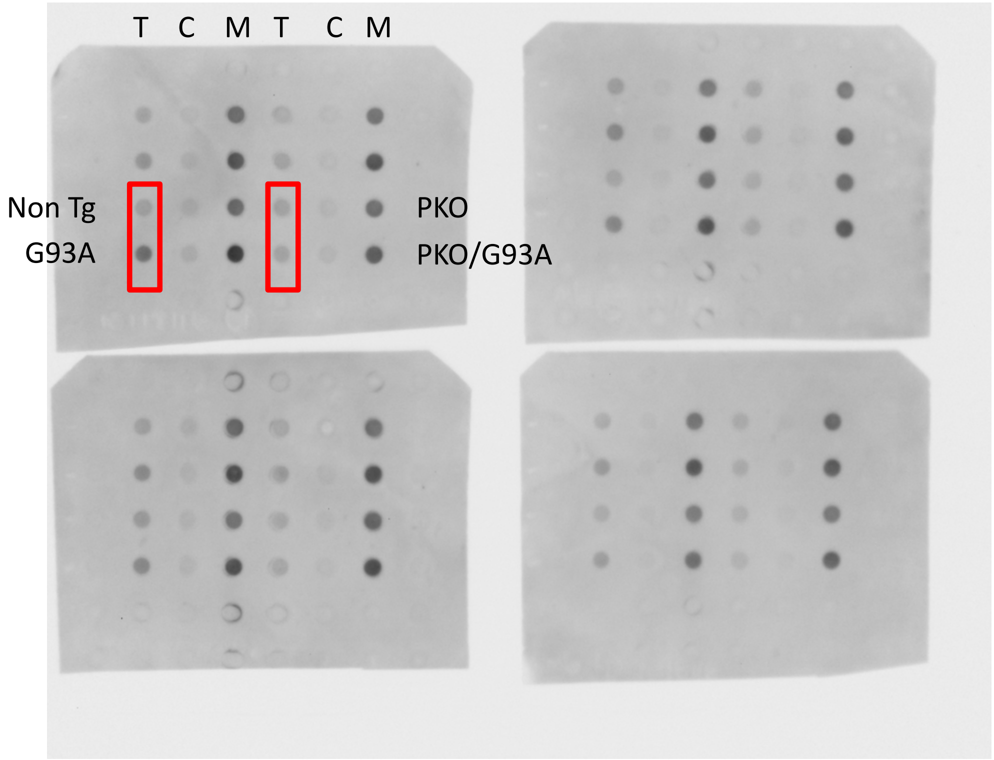

Palomo GM et al. Figure 4

p62 staining of lumbar spinal cords

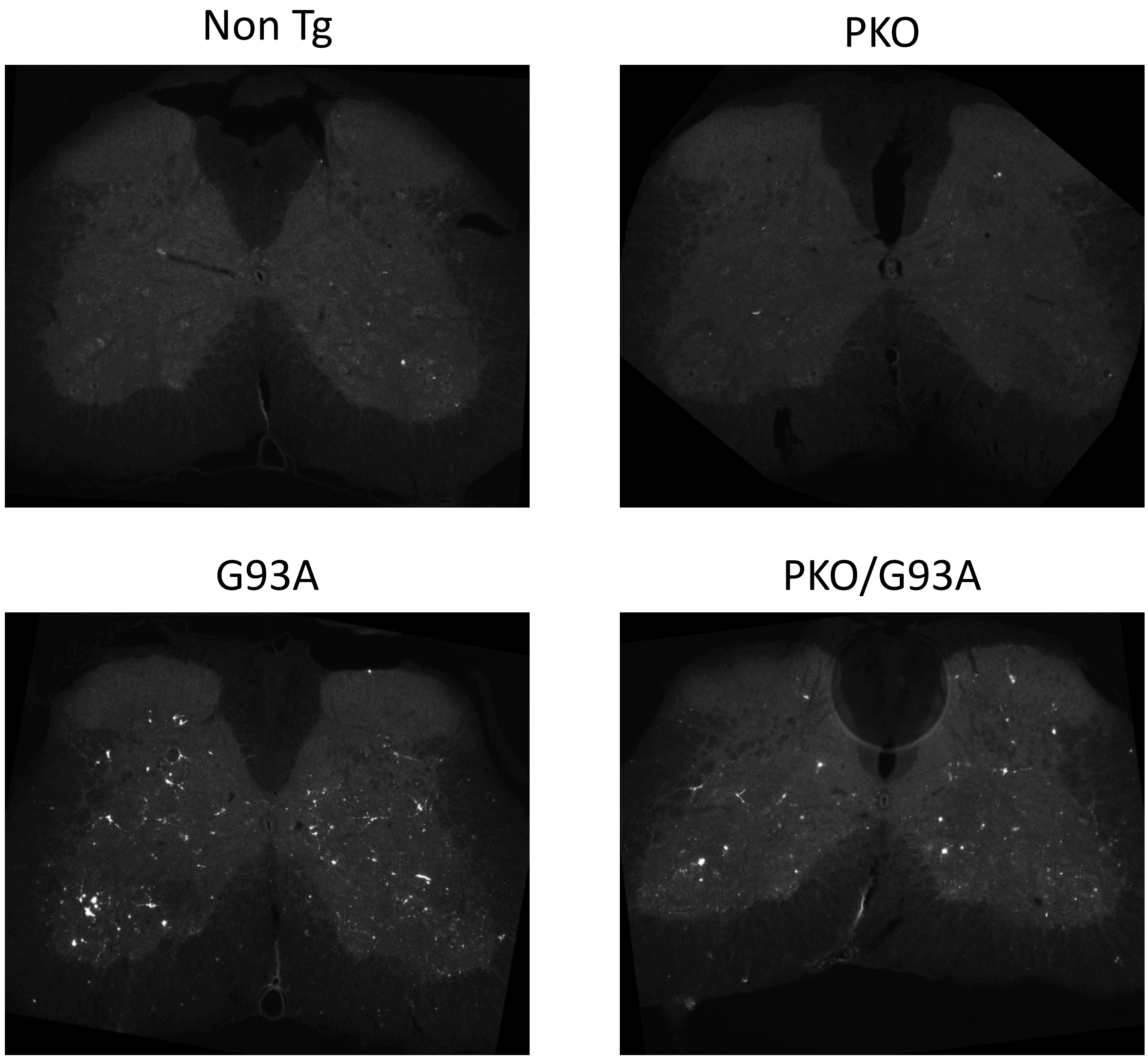

Supplement: Supplementary file 9 — Source Data for Figure 4 [file EMMM-10-e8888-s007.pdf]
