# Supplementary material for: Parkin is a disease modifier in the mutant SOD1 mouse model of ALS
Source: EMBO Mol Med. 2018 Aug 20;10(10):e8888. doi: 10.15252/emmm.201808888 (PMC6180298; doi:10.15252/emmm.201808888)

Palomo GM et al. Figure 8

Developed with anti-Miro1

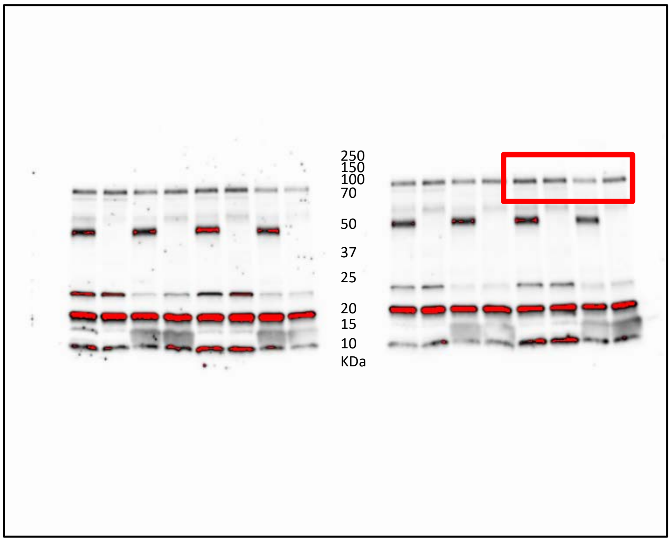

Developed with anti-β-actin

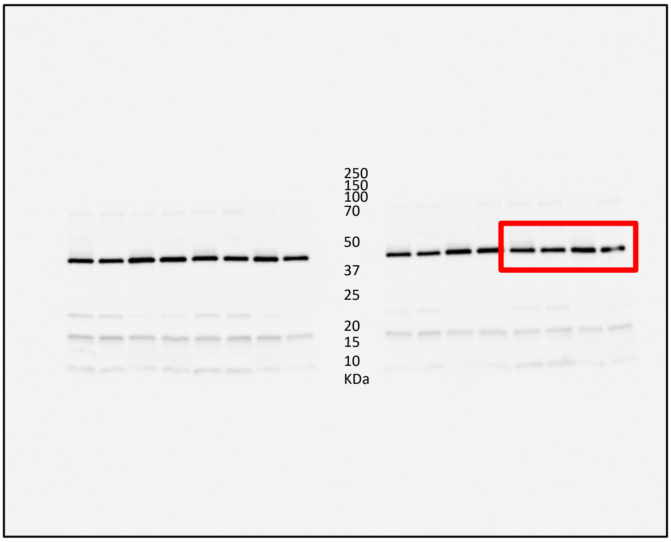

Developed with anti-Mfn2

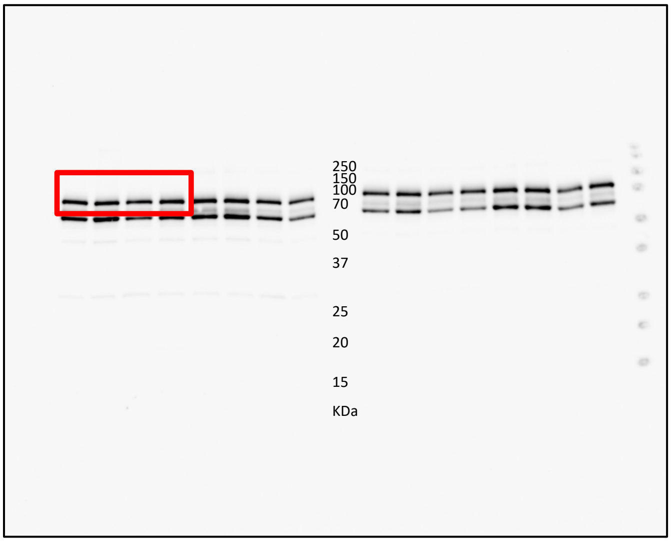

Developed with anti-β-actin

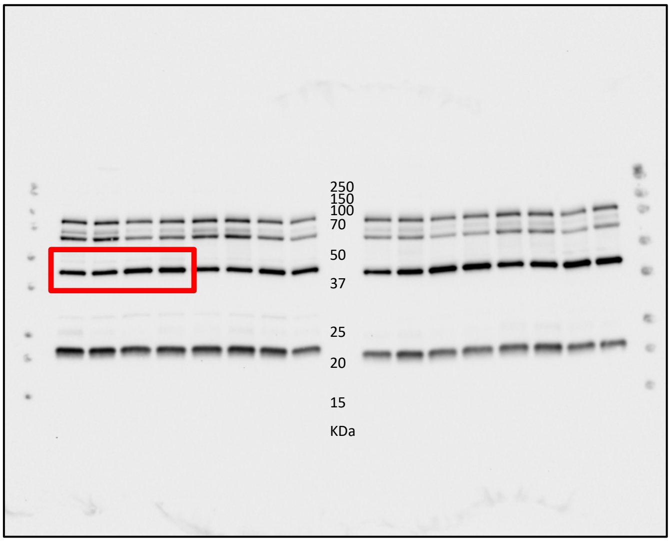

Palomo GM et al. Figure 8

Developed with anti-Apu2

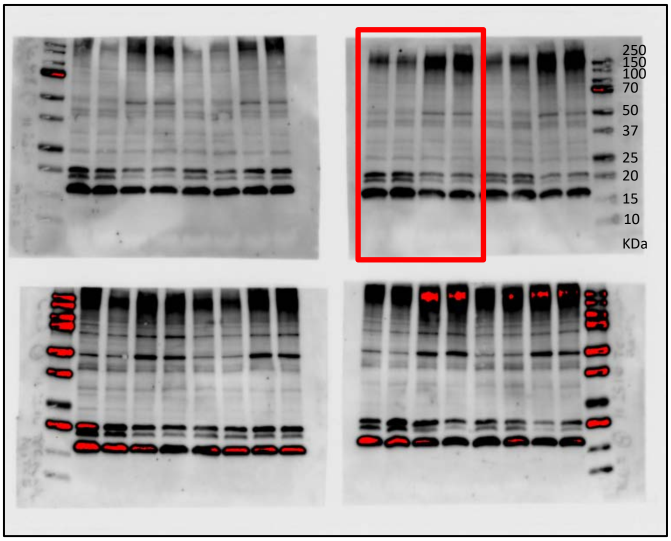

Developed with anti-CS

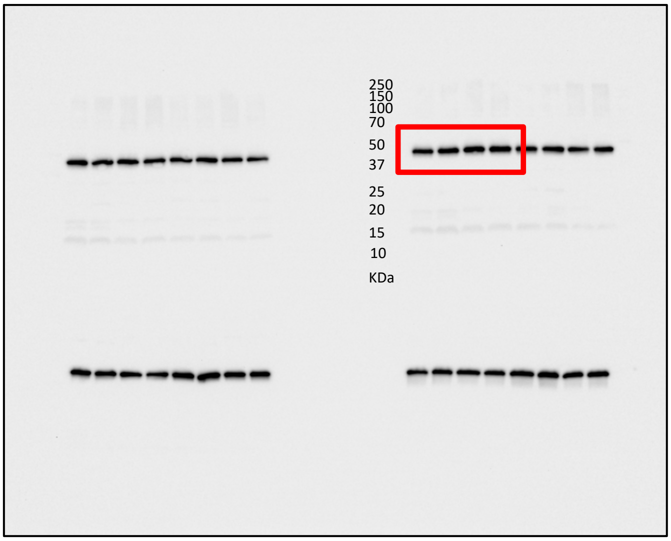

Developed with anti-Apu3

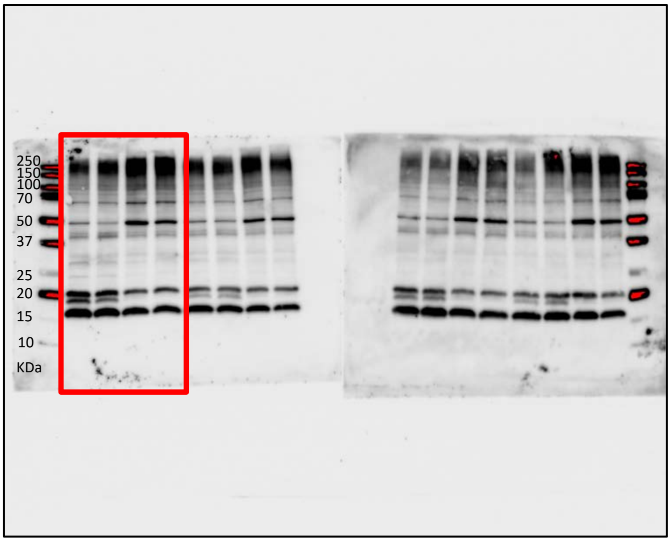

Developed with anti-CS

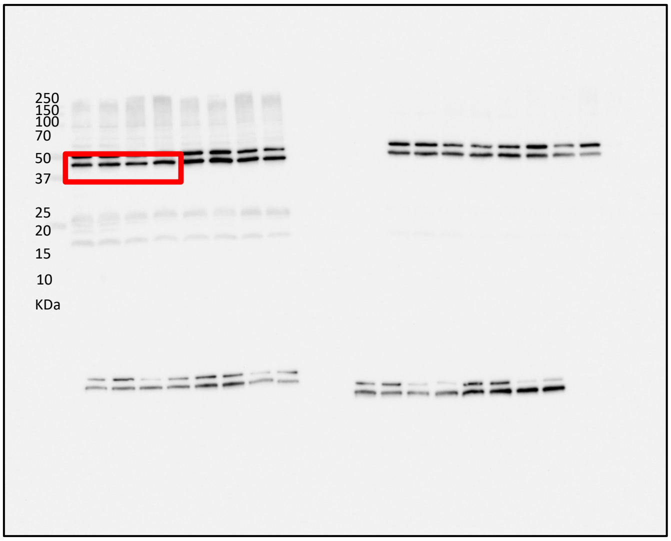

Palomo GM et al. Figure 8

Developed with anti-LC3

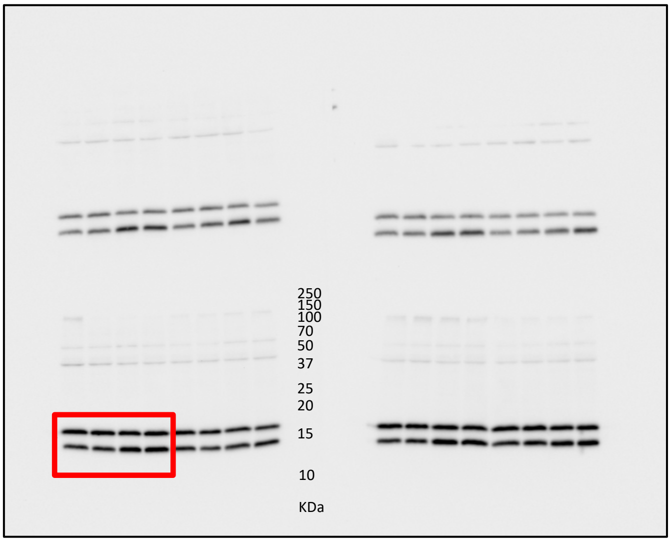

Developed with anti-Complex V

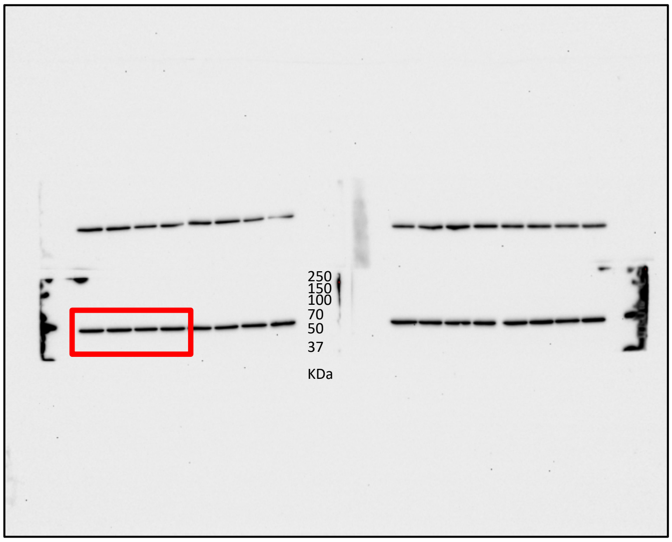

Palomo GM et al. Figure 8

Developed with anti-Mul1

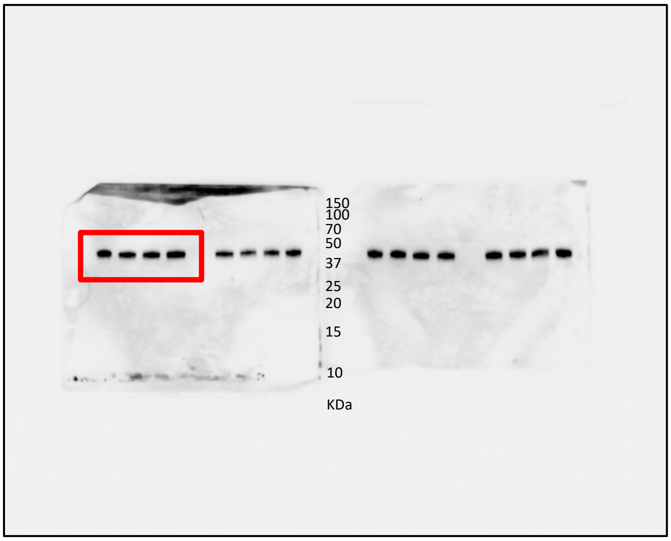

Developed with anti-MARCH5

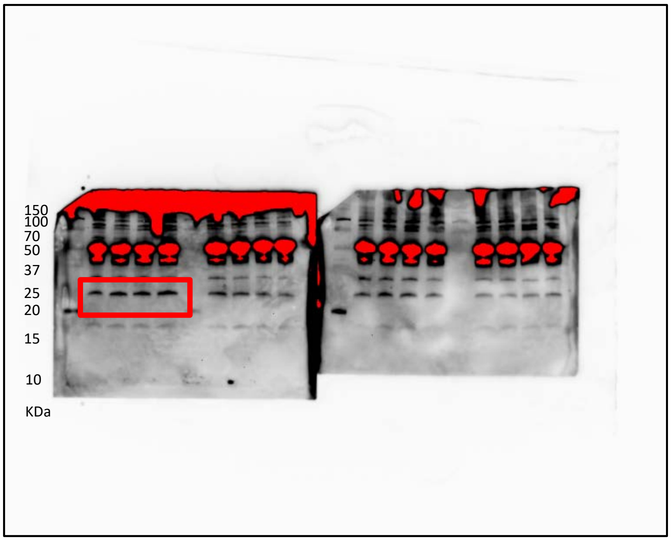

Developed with anti-Complex V

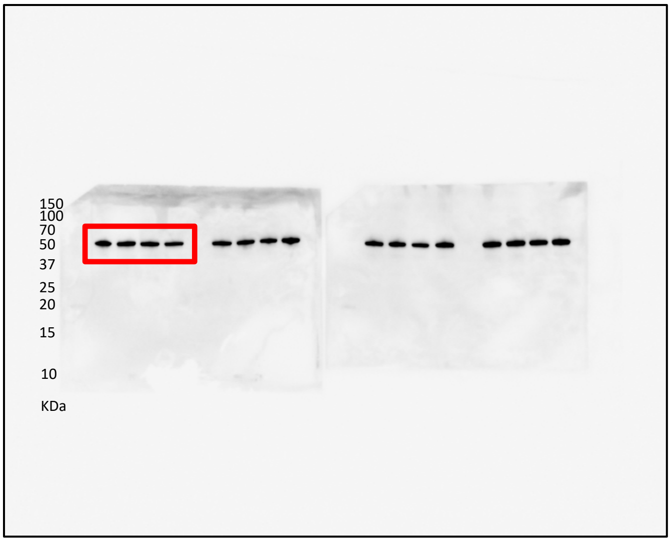

Supplement: Supplementary file 13 — Source Data for Figure 8 [file EMMM-10-e8888-s011.pdf]
